# Supplementary material for: PDLIM2 Suppression Inhibit Proliferation and Metastasis in Kidney Cancer
Source: Cancers (Basel). 2021 Jun 15;13(12):2991. doi: 10.3390/cancers13122991 (PMC8232651; doi:10.3390/cancers13122991)
Supplement: Supplementary file 1 [file cancers-13-02991-s001.zip › Supplement Table S1.pdf]

Supplement Table S1. Oligonucleotide sequences for RT-qPCR and shRNA cloning

Primer sequences for Reverse transcription	
18S_rRNA_RT_Fwd	TTCGTATTGAGCCGCTAGA
18S_rRNA_RT_Rev	CTTTCGCTCTGGTCCGTCTT
hPDLIM2_RT_Fwd	CCTCTCCTGCTGCCTTACAG
hPDLIM2_RT_Rev	GGAAGAGAGGACCAGGAGGT
hCDH1_RT_Fwd	TCGGACCAAGGACAAGTACC
hCDH1_RT_Rev	ATCTTCACCTGCCGTTCAGT
hCDH2_RT_Fwd	GACAATGCCCCTCAAGTGTT
hCDH2_RT_Rev	CCATTAAGCCGAGTGATGGT
hVIM_RT_Fwd	GAGAACTTTGCCGTTGAAGC
hVIM_RT_Rev	GCTTTCTGTAGGTGGCAATC
Oligonucleotide sequences for shRNA lentiviral vector cloning	
Upper_TurboGFP_shRNA	CCGGCGTGATCTTCACCGACAAGATCTCGAGATCTTGT CGGTGAAGATCACGTTTTT
Bottom_TurboGFP_shRNA	AATTAAAAACGTGATCTTCACCGACAAGATCTCGAGA TCTTGTCGGTGAAGATCACG
Upper_PDLIM2(h)_shRNA01	CCGGAGACATAATCGTGGCCATCAACTCGAGTTGATG GCCACGATTATGTCTTTTTTT
Bottom_PDLIM2(h)_shRNA01	AATTAAAAAAGACATAATCGTGGCCATCAACTCGAGT TGATGGCCACGATTATGTCT
Upper_PDLIM2(h)_shRNA02	CCGGACATAATCGTGGCCATCAACTCTCGAGAGTTGAT GGCCACGATTATGTTTTTTT
Bottom_PDLIM2(h)_shRNA02	AATTAAAAAAACATAATCGTGGCCATCAACTCTCGAGA GTTGATGGCCACGATTATGT
