# Supplementary figures and images for: PDLIM2 Suppression Inhibit Proliferation and Metastasis in Kidney Cancer
Source: Cancers (Basel). 2021 Jun 15;13(12):2991. doi: 10.3390/cancers13122991 (PMC8232651; doi:10.3390/cancers13122991)

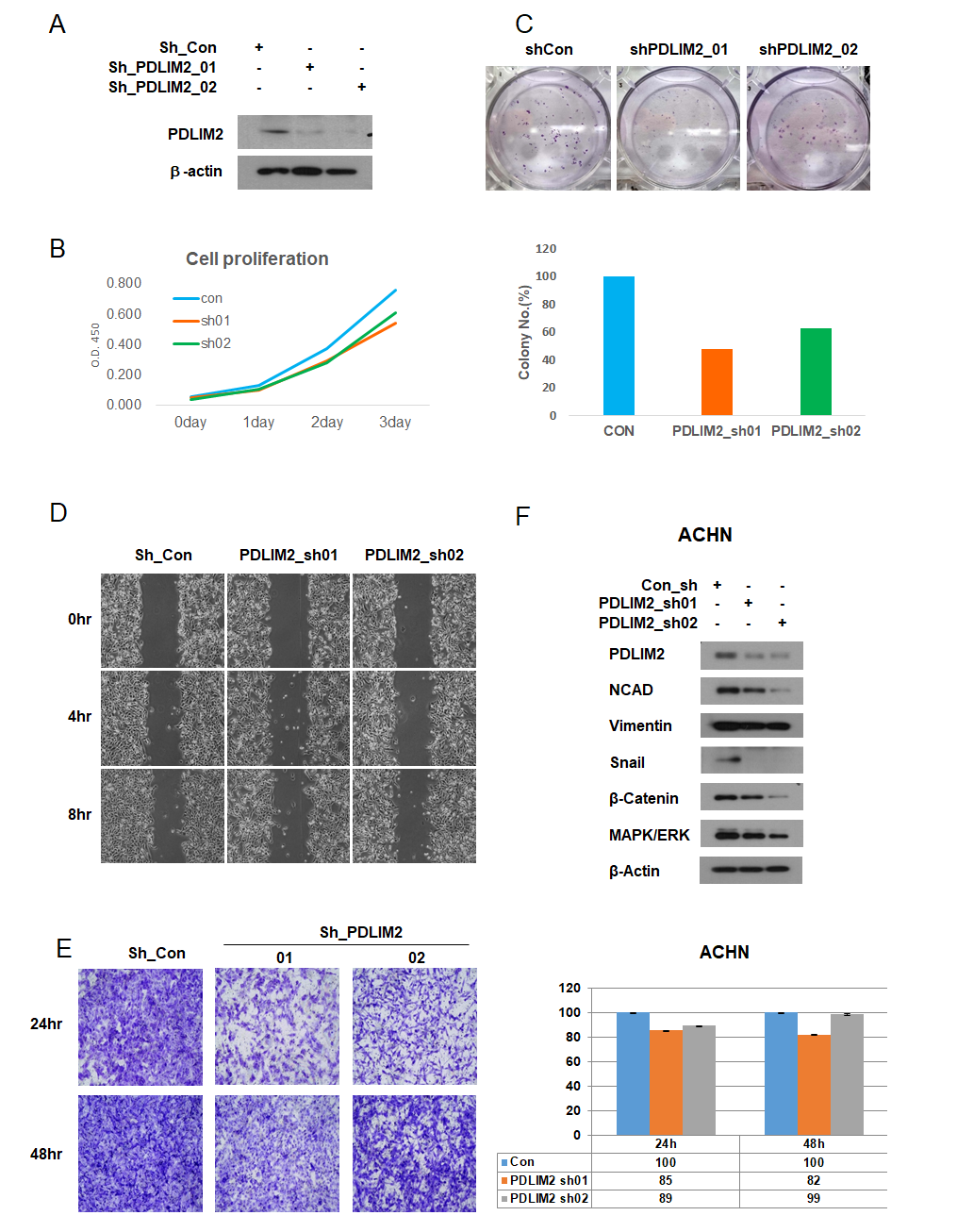

Supplement: Supplementary file 1 [file cancers-13-02991-s001.zip › Supple Figure S1.tif]
